# Toxicities of Immunotherapy for Small Cell Lung Cancer

**DOI:** 10.3389/fonc.2021.603658

**Published:** 2021-05-31

**Authors:** Yang Fu, Yue Zheng, Pei-Pei Wang, Zhen-Yu Ding

**Affiliations:** Department of Biotherapy, Cancer Center, West China Hospital, West China Medical School, Sichuan University, Chengdu, China

**Keywords:** small cell lung cancer, immune-related adverse events, neuromuscular toxicity, immune checkpoint inhibitors, death

## Abstract

Small cell lung cancer (SCLC), composing 15–20% of lung cancer, is a fatal disease with extremely poor prognosis. In the past two decades, etoposide platinum doublet chemotherapy remained the only choice of therapy, with disappointing overall survival ≤1 year for the metastatic disease. Novel treatments including immunotherapy are urgently needed and extensively explored. Recently, in two phase III trials, atezolizumab and durvalumab were shown to bring survival benefit to patients. While immunotherapy brings better outcome, it is accompanied by adverse events different from traditional treatments. Although these immune-related adverse events (irAEs) are generally mild and can be managed, some irAEs (myocarditis, pneumonitis) may be severe and even life-threatening. Accompanying with the increasing application of immunotherapy in clinical practice, the irAEs should not be overlooked. In this review, the irAEs profile in clinical trials of immunotherapy for SCLC will be summarized, also its unique features compared with irAEs in other malignancies will be explored. This review may be helpful for the appropriate clinical use of immunotherapy for SCLC.

## Introduction

Small cell lung cancer (SCLC) is a fatal disease, with a 5-year survival less than 7% (1, 2). Platinum doublet chemotherapy, usually combined with etopside, remains the standard-of-care for decades (3–5). Patients have a high initial response rates of 60%, while most relapse within 6 months and decease within 10 months (5–7).

Immune checkpoint helps to maintain the immune stability, while during carcinogenesis it is hijacked by tumors to evade immune surveillance. Immune checkpoint inhibitors (ICIs) including antibodies against cytotoxic T-lymphocyte antigen 4 (CTLA-4) or programmed cell death 1 (PD-1)/programmed cell death ligand 1 (PD-L1) act to reverse the immunosuppression imparted by tumor cells, either by blocking CTLA-4 pathway or interrupting the interaction between PD-1 and PD-L1 (8, 9). ICI has been widely used in a variety of malignancies, including non-small cell lung cancer, melanoma, triple-negative breast cancer, and non-Hodgkin lymphoma etc. (8, 10–12). Especially in SCLC which has a notorious reputation of poor prognosis, PD-L1 inhibitors including atezolizumab and durvalumab show promising efficacy (9, 13).

While immunotherapy brings better outcome, it is accompanied by adverse events different from traditional treatments. Mounted immune response is directed to not only tumor, but also normal tissues and causes immune-related adverse events (irAEs) (14). Although these irAEs are generally mild and can be managed, some irAEs (myocarditis, pneumonitis) may be severe and even life-threatening (15, 16).

Recently, in two phase III trials (IMPower 133 and CASPIAN), atezolizumab and durvalumab were shown to bring survival benefit to patients (9, 13). Accompanying with the increasing application of immunotherapy in clinical practice, the irAEs should not be overlooked. In SCLC, due to the poor life expectance, also the high incidence of neurological complications, it is intriguing to ask whether the irAEs would be different from other tumors. This review provided a brief summary of irAEs from published clinical trials in the field of SCLC treatment.

## Overview of SCLC

SCLC is a distinct form of lung cancer, with dominant component of neuroendocrine tumor cells, and early and frequent distant metastases (17). Mutations in p53 gene (TP53) and retinoblastoma1 gene (RB1) are universal genetic events in SCLC (18). Studies also showed although SCLC harbors a high tumor mutational burden, tumor infiltrating lymphocytes are scarce in the microenvironment (19). Neither SCLC tends to express PD- L1, as it is found ≤20% tumor cells express PD-L1 (>1%) (20, 21).

Etoposide plus platinum combination chemotherapy is recommended for metastatic SCLC patients (extensive stage, ES). For those in the limited stage (LS, non-metastatic), chest radiation at a dose of 45 Gy administered in 1.5 Gy fractions twice-daily for 30 days with chemotherapy, followed by prophylactic cranial irradiation is recommended (22). For relapsed or platinum-refractory SCLC, topotecan was the only approved drug by FDA in second-line treatment (23). Meanwhile, clinical trials on inhibitors of PARP, EZH2, WEE1, DLL3, and Aurora kinase etc. are all actively ongoing at this time, which is beyond the scope of this review (24). Here, we restrict our focus on the clinical data of the immunotherapy for SCLC.

## Data Acquisition

All relevant articles are identified by using the keywords “small cell lung cancer,” “SCLC,” “immunotherapy,” “CTLA-4,” “PD-1,” “PD-L1,” “clinical trial” on Pubmed, clinicaltrials.gov, Embase and Web of science. Abstracts and presentation were also reviewed from major conference including ASCO (https://www.asco.org/) and ELCC (https://www.esmo.org/) from 2015 to 2020.The literature or abstract was viewed, and those with only protocol design or lack of AEs results were excluded. Finally, fifteen studies involving ICIs for SCLC therapy with full description of the AEs were selected.

## Landscape of Immunotherapy for SCLC

### First Line

The first one being tested was ipilimumab, a fully human monoclonal antibody for CTLA-4. Following a successful phase II study (NCT00527735), a phase III trial (CA184-156) investigated the efficacy and safety of ipilimumab combined with chemotherapy (25, 26). However, the addition of ipilimumab failed to demonstrate any improvement in neither OS, ORR, nor duration of response. IMPower133 was a phase III trial to investigate the efficiency of atezolizumab (a humanized monoclonal PD-L1 antibody) combined with chemotherapy. The combination regimen showed benefit in both PFS (5.2 m *vs* 4.7 m) and OS (12.3 m vs 10.3 m) (9). A similar good outcome was also achieved by durvalumab, another high-affinity human IgG1 monoclonal antibody for PD-L1. In the phase III CASPIAN study, the combination of durvalumab and chemotherapy achieved an OS of 13.3 m (13). The results of PD-1 antibodies seemed less favorably. KEYNOTE-604 was a phase III trial to investigate the efficacy of pembrolizumab (a humanized monoclonal IgG4 antibody) in ES-SCLC patients (27). The results showed that pembrolizumab significantly improves PFS, while OS narrowly had significant difference. Nivolumab is another monoclonal antibody for PD-1. A phase II randomized study (EA5161) evaluated the combination of nivolumab with EP for the ES-SCLC patients. Preliminary results were reported in ASCO 2020, nivolumab significantly improved PFS (5.5 and 4.7 m, p = 0.047) in treated population while OS was no statistical difference (11.3 and 8.5 m, p = 0.14) (28).

### Maintenance

A phase III study (CHECKMATE-451) tested either nivolumab monotherapy, or nivolumab plus ipilimumab, or placebo as maintenance therapy after platinum-based first-line chemotherapy. However, nivolumab has a shorter OS compared with placebo (29). Another phase II, single-arm trial (NCT02359019) studied pembrolizumab as maintenance therapy. The 1-year PFS and OS rates were only 13 and 37% respectively (30).

### Second Line

Salvage therapy for the relapsed SCLC is more difficult. At least two randomized controlled trial tested the efficacy of immunotherapy. In IFCT-1603 study, atezolizumab monotherapy was compared with topotecan or re-induction of initial chemotherapy (31). A phase III trial CHECKMATE-331 investigated the efficacy and safety of nivolumab monotherapy in the second line of therapy (32). Both trials demonstrated no superiority of immunotherapy over traditional chemotherapy.

Monotherapy seems inappropriate, and following studies tested combination therapy. In a multi-center, single arm, phase II study (NCT02551432), pembrolizumab was combined with chemotherapy drug paclitaxel (33). In another phase II study (NCT02484404), Durvalumab was tested in combination with olaparib (PARP inhibitors) (34). The preliminary reports of these small sample sized showed promising results.

### Third Line or Later

Some early, small-scale studies were performed in these very late-staged patients. Nivolumab was the first ICIs approved by FDA for third-line therapy of SCLC, based on the results of CHECKMATE-032 in 2016 (35). KEYNOTE-028 (NCT02054806) and KEYNOTE-158 studies both tested pembrolizumab in the third line therapy. Based on the results, pembrolizumab monotherapy was approved to SCLC in third-line or later (36, 37).

## Toxicities

### Ipilimumab

In the phase II study (NCT00527735), ipilimumab plus chemotherapy led to higher frequency of AE, either any grade (49 and 43%) or ≥grade 3 (G3, 46 and 30%) AE than chemotherapy alone. Common severe irAEs included G4 diarrhea (n = 1), G3 colitis (n = 1), G4 hepatitis (n = 2), and death (n = 1) attributed to hepatotoxicity (25). In the following phase III study (CA184-156), the combination also had higher incidence of irAEs of all grade (57% in ipilimumab group, and 28% in control) or ≥G3 (20 and 2%). Gastrointestinal and skin toxicity (34 and 29%) were the most common irAEs in ipilimumab group. Endocrine irAEs occurred in 10% of patients in the ipilimumab group including hypothyroidism (3%), hyperthyroidism (2%), hypophysitis (1%), and adrenal insufficiency (1%). Two deaths due to colitis (n = 1) and ulcerative colitis (n = 1) were reported. The incidence of nervous system irAEs was 4% which involved 2% of peripheral sensory neuropathy (26).

### Atezolizumab

In IMPower133 study, the incidence of AEs was 39.9% in the atezolizumab group and 24.5% in the control group. The most common irAEs was rash (18.7%), hypothyroidism (12.6%), hepatitis (7.1%), and hyperthyroidism (5.6%). The less frequent (≤5%) of irAEs were pneumonitis (2.0%), colitis (1.5%), rhabdomyolysis (1.0%), severe cutaneous reaction (1.0%), pancreatitis (0.5%), nephritis (0.5%), hypophysitis (0.5%), and diabetes mellitus (0.5%). Severe irAEs (≥G3) were rash (2%), hepatitis (1.5%), infusion-related reaction (2%), and colitis (1%) (9). In IFCT-1603 study, the incidence of AE, including 12.5% musculoskeletal or connective tissue disorders, 18.8% gastrointestinal disorders, 4.2% hepatitis, 4.2% colitis, 6.3% arthralgia, 2.1% hyperthyroidism and 2.1% hypothyroidism. No≥G3 irAE was reported (31).

### Durvalumab

In CASPIAN study, three groups were enrolled, including durvalumab and chemotherapy, combo immunotherapy durvalumab and tremelimumab with chemotherapy, and chemotherapy. The incidences of ≥G3 AEs were 62.3, 70.3, and 62.8% in each of these groups. G5 AEs were 4.9, 10.2 and 5.6%, respectively. For G3–4 irAEs, the incidence was 5% in durvalumab group and ≤1% in control group, and it was 20 and 3% for any grade. Endocrine-related adverse events were the most common irAEs including hypothyroidism (9%), hyperthyroidism (5%), thyroiditis (4%), type 1 diabetes mellitus (T1DM, 2%), rash (2%), adrenal insufficiency (<1%). The incidence of immune-related pneumonitis was 3% of all grades and 1% of G3–4. There were also reports of immune-related colitis, pancreatic events, and hepatic events. Two immune-related deaths due to hepatotoxicity (n = 1) and pneumonitis (n = 1) were reported (13).

The phase II study (NCT02484404) was an exploratory study. In this study, nine patients (45%) had G3–4 TRAEs including anemia, lymphopenia, thrombocytopenia, and hypophosphatemia. In five patients’ hypothyroidism was observed attributed to immunotherapy (34).

### Pembrolizumab

In the 1st line setting (KEYNOTE-604), when pembrolizumab used with chemotherapy, the incidence of irAEs (any grade) was 53%, compared with 84% in the control group. Hypothyroidism (10.3%), hyperthyroidism (6.7%), and pneumonitis (4%) were the most common. G3 irAEs occurred in only 7.2% of patients, and no G4–5 irAEs occurred (27). The only maintenance therapy study (NCT02359019) reported three categories of irAEs, rash (n = 8), hypothyroidism (n = 4), T1DM with diabetic ketoacidosis (n = 1) (30). In late lines of pembrolizumab monotherapy (KEYNOTE-028), the most frequent AEs were arthralgia, asthenia, and rash (n = 4 each) as well as diarrhea and fatigue (n = 3 each). Only two patients experienced G3 AE. One had G3 bilirubin elevation, and the other was a lethal case of colitis concurrent with G3 bilirubin elevation. Another similar study (KEYNOTE-158) reported AE of any grade and G3–5 were 33.7 and 5.1%, respectively. Most common irAEs included hypothyroidism (12.1%), hyperthyroidism (6.5%), severe skin reactions (2.8%), adrenal insufficiency, nephritis, pancreatitis, and pneumonitis (1.9% each). G3 AE occurred in six patients, mostly manageable, and no fatal irAE was reported (36, 37). In an early-phase exploratory study (NCT02551432), AEs occurred in all patients. Pneumonia (19.2%), T1DM (7.7%), rush (7.7%), and hypothyroidism (3.9%) were among the most common irAEs. Four patients discontinued treatment (33).

### Nivolumab

In the study CheckMate-331, TRAEs of all grade (≥G3) occurred in 55% (14%) of nivolumab group, and 90% (73%) of chemotherapy group. There were five treatment-related death, two with nivolumab and three with chemotherapy. The incidences of irAEs (all grade) of endocrine, skin, gastrointestinal, liver, lung and kidney were 12, 11, 7, 5, 1 and <1% respectively (32). In study CheckMate-032, **s**kin toxicity (any grade, 21.1%) was the most common. Other irAEs including endocrine, gastrointestinal, hepatic, pulmonary and renal toxicity were 9.2, 6.4, 4.6, 1.8 and 0.9% respectively. The incidence of G3–4 pneumonitis, rash, aspartate aminotransferase increase was 1.8, 0.9, and 0.9%, respectively. One immune-related encephalitis (grades 3–4) was reported. One death due to checkpoint inhibitor pneumonitis was noted (35). While in study CheckMate-451, the most frequently occurred serious AEs was pneumonitis (3.8%). Other serious included colitis (3.6%), endocrine (2.5%), hepatitis (0.7%), and nervous system (3.7%). Myocarditis was reported in two cases (0.7%) in group. AEs in nervous system were encephalitis (n = 2), myasthenia gravis (n = 1), and Guillain–Barré syndrome (n = 1). There were eight treatment-related deaths in the nivolumab group versus one in the control group (29). In study EA5161, the incidence of grade 3/4 TRAEs was 77% *vs* 62%. Treatment-related fatal adverse events were similar in the two groups (n = 9 and 7) (28).

## Discussion

**Figure 1:**
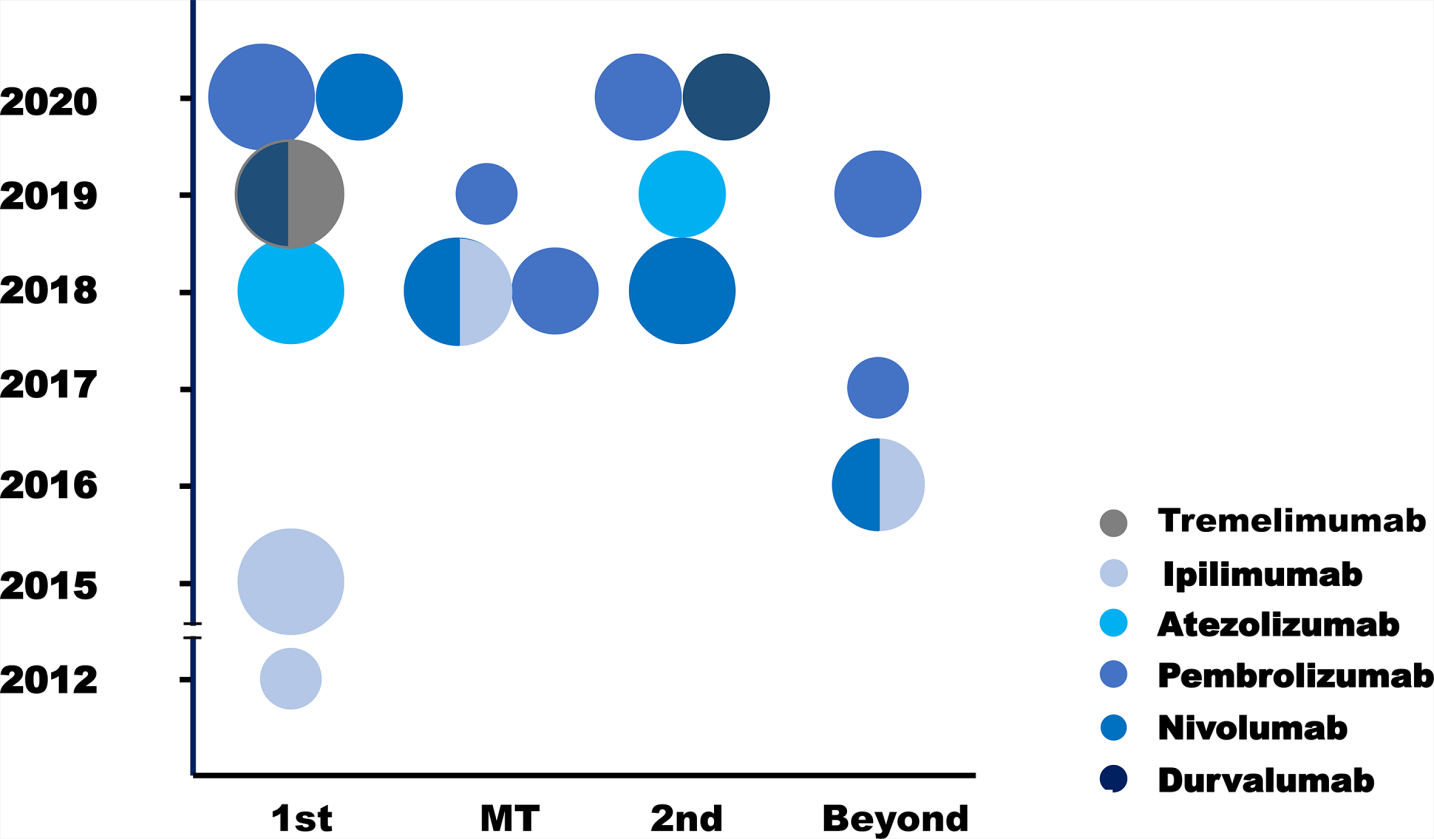
Clinical trials of ICIs in SCLC. Each circle standed for one trial. The different circle areas represented phase I, phase II and phase III trials respectively, and different color meant different drugs. Y axis stood for the year of the publication of each trial, and X trials was the clinical situation where each trial was performed. 1st, first line; MT, maintenance therapy; 2nd, second line; Beyond, later line.

This review summarized 15 trials in SCLC immunotherapy, including phase III (n = 5) and phase I/II trials (n = 10, **Figure 1**). Among them, IMpower133, CASPIAN, CA184-156, KEYNOTE-604 and EA5161 evaluated the efficacy of atezolizumab, durvalumab, ipilimumab, pembrolizumab, or nivolumab, when combined with chemotherapy. CheckMate-331 and IFCT-1603 tested the efficacy of nivolumab and atezolizumab monotherapy in 2nd-line. Six trials investigated efficacy and safety of ICIs in later-line or maintenance treatment. Most trials were performed in ICIs combined with chemotherapy. More studies are ongoing (**Table 1**).

**Table 1:** Ongoing trial with PD-1/PD-L1 inhibitors in the treatment of small cell lung cancer.

Trial		Treatment	Phase	Intervention	Population	Patients	Therapy	Status
NCT02580994	REACTION	First line	II	RCT	ES-SCLC	125	Pembrolizumab + EC/EP *vs* EC/EP	Recruiting
NCT02402920		First line	I	Parallel	SCLC	80	Pembrolizumab + Concurrent Chemo/Radiotherapy	Recruiting
NCT02963090		Second line	II	RCT	Relapsed	98	Pembrolizumab *vs* Topotecan	Active
NCT03371979		Second line	I/II	Single arm	Relapsed	84	Pembrolizumab + Pegzilarginase(AEB1102)	Active
NCT04358237	LUPER	Second line	I/II	Single arm	Relapsed	42	Pembrolizumab + Lurbinectedin (PM01183)	Not yet recruiting
NCT03253068		Second line	II	Single arm	Relapsed	25	Pembrolizumab + Amurubicin	Recruiting
NCT04173325		Second line	I	Single arm	Relapsed	10	Nivolumab + Irinotecan	Recruiting
NCT03406715		Second line	II	Multicohort	SCLC	40	Nivolumab+ Ipilimumab+ Dendritic Cell p53 Vac	Recruiting
NCT03083691	BIOLUMA	Second line	II	Multicohort	Relapsed	106	Nivolumab + Ipilimumab	Recruiting
NCT03670056		Second line	II	Single arm	Relapsed	40	Nivolumab + Ipilimumab	Recruiting
NCT03728361		Second line	II	Multicohort	Relapsed	53	Nivolumab + Temozolomide	Recruiting
NCT03575793		Second line	I/II	Parallel	Relapsed	55	Nivolumab + Ipilimumab + Plinabulin	Recruiting
NCT03662074		Second line	II	Single arm	Relapsed	14	Nivolumab + Gemcitabine	Active
NCT02247349		Second line	I/II	Parallel	Relapsed	172	BMS-986012 + Nivolumab *vs* BMS-986012	Active
NCT03325816		Maintenance	I/II	Single arm	ES-SCLC	9	Nivolumab + Lutathera	Active
NCT03958045		Maintenance	II	Single arm	SCLC	36	Nivolumab + Rucaparib	Recruiting
NCT02046733	STIMULI	Maintenance	II	Parallel	LS-SCLC	264	Nivolumab + Ipilimumab After Chemo-radiotherapy	Active
NCT04189094		First line	II	RCT	LS-SCLC	140	Sintilimab + EC/EP + RT *vs* EC/EP + RT	Not yet recruiting
NCT04192682		Second line	II/III	Single arm	Relapsed	40	Sintilimab + Anlotinib after Chemo-radiotherapy	Not yet recruiting
NCT04055792		Beyond	II	RCT	ES-SCLC	52	Sintilimab + Anlotinib *vs* Anlotinib	Recruiting
NCT03983759		Maintenance	II	Single arm	ES-SCLC	40	Sintilimab After Chemotherapy + R-CIK	Recruiting
NCT04449861	ORIENTAL	First line	IIIb	Single arm	ES-SCLC	300	Durvalumab + EC/EP	Not yet recruiting
NCT03509012	CLOVER	First line	I	Multicohort	SCLC	360	Durvalumab ± Tremelimumab + EC/EP + Radiotherapy	Active
NCT04361825		Second line	II	Single arm	Relapsed	45	Durvalumab + AZD6738	Recruiting
NCT02701400		Second line	II	Parallel	Relapsed	18	Durvalumab + Tremelimumab ± RT	Active
NCT02937818		Second line	II	Parallel	Refractory*	72	Durvalumab + Tremelimumab *vs* AZD1775 + carboplatin *vs* AZD6738 + Olaparib	Active
NCT04314297		Maintenance	II	Single arm	ES-SCLC	33	Durvalumab + Anlotinib after Chemo-radiotherapy	Not yet recruiting
NCT04472949		Maintenance	II	Single arm	ES-SCLC	46	RT+ Durvalumab after Durvalumab + EC	Not yet recruiting
NCT03585998		Maintenance	II	Single arm	LS-SCLC	51	Durvalumab after Chemo-radiotherapy + Durvalumab	Active
NCT03703297	ADRIATIC	Maintenance	III	RCT	LS-SCLC	600	4Durvalumab + 4Placebo;Durvalumab	Recruiting
							4Durvalumab + 4Tremelimumab;Durvalumab	
							4 Placebo; Placebo	
NCT03923270		Maintenance	I	Parallel	ES-SCLC	54	RT followed by Durvalumab or Durvalumab + Tremelimumab or Olaparib	Recruiting
NCT04256421	SKYSCRAPER-02	First line	III	RCT	ES-SCLC	400	Atezolizumab + EC+ Tiragolumab *vs* Atezolizumab + EC	Recruiting
NCT03041311		First line	II	RCT	ES-SCLC	105	Atezolizumab + EC + Trilaciclib(G1T28) *vs* Atezolizumab + EC	Active
NCT03540420		First line	II	RCT	LS-SCLC	212	Atezolizumab *vs* standard care after Chemo-radiotherapy	Recruiting
NCT04028050	MAURIS	First line	IIIb	RCT	ES-SCLC	150	Atezolizumab + EC	Recruiting
NCT04422210		First line	Ib	Single arm	ES-SCLC	62	Venetoclax + Atezolizumab + EC	Recruiting
NCT03262454		Second line	II	Single arm	Relapsed	35	Radiotherapy Followed by Atezolizumab	Recruiting
NCT03059667		Second line	II	RCT	SCLC	70	Atezolizumab *vs* Topotecan/Etoposide/Carboplatin	Active
NCT04402788	RAPTOR	Second line	II/III	RCT	ES-SCLC	324	Atezolizumab + RT *vs* Atezolizumab	Not yet recruiting
NCT04308785		Maintenance	II	RCT	LS-SCLC	242	Atezolizumab + EC/EP+ radiotherapy *vs* EC/EP + radiotherapy	Not yet recruiting
NCT04462276	TREASURE	Maintenance	II	RCT	ES-SCLC	104	Atezolizumab + RT *vs* Atezolizumab after Atezolizumab + EC	Not yet recruiting
NCT04373369		Maintenance	II	Single arm	ES-SCLC	33	Atezolizumab + Vorolanib	Not yet recruiting
NCT03811002		First line	II/III	RCT	LS-SCLC	506	Atezolizumab + EC/EP + RT *vs* EC/EP + RT	Recruiting
NCT04418648		consolidation	II	RCT	LS-SCLC	170	Toripalimab *vs* Observation	Not yet recruiting
NCT04363255		Maintenance	II	Single arm	ES-SCLC	20	EC/EP followed by Toripalimab + Anlotinib	Not yet recruiting
NCT04012606		First line	III	RCT	ES-SCLC	420	Toripalimab(JS001) + EC/EP *vs* EC/EP	Recruiting

*Platinum Refractory ES-SCLC.

**Figure 2:**
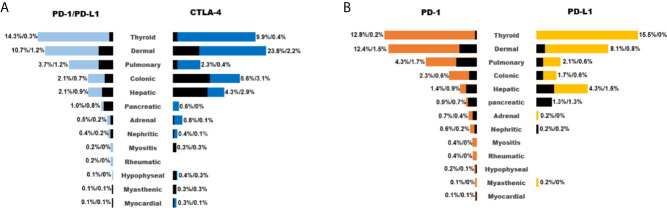
List of common irAEs for different ICIs (**A**: PD-1/PD-L1 inhibitors vs CTLA-4 inhibitors; **B**: PD-1 inhibitors vs PD-L1 inhibitors). Colored and black bar indicated the occurrence of irAEs of any grade and ≥grade 3.

When all the 15 trials combined for analysis, PD-1/PD- L1 inhibitors had a better tolerance than CTLA-4 inhibitors (**Figure 2A**). Dermal events (23.8%), colitis (5.6%), hepatitis (4.3%), hypophysitis (0.4%), myasthenic (0.3%), and myocarditis (0.3%) were more common with CTLA-4 inhibitors, whereas pneumonitis (3.7%), thyroid events (14.3%), pancreatic events (1.0%), and rheumatic events (0.2%) were more common with PD-1/PD- L1 inhibitors. It was also interesting to observe the difference of toxicities between PD-1 and PD-L1 inhibitors. Generally, the rate of irAE by PD-L1 inhibitors was lower than that of PD-1 inhibitors, including pneumonitis (4.3% *vs* 2.1%), dermal events (12.4% *vs* 8.1%), colitis (2.3% *vs* 1.7%), adrenal insufficiency (0.7% *vs* 0.2%), nephritis (0.6% *vs* 0.2%), myositis (0.4% *vs* 0), rheumatic disease (0.4% *vs* 0), hypophysitis (0.2% *vs* 0), and myocarditis (0.1% *vs* 0, **Figure 2B**).

In CheckMate-451 trial, the frequency of irAEs of the nivolumab plus ipilimumab group was higher than that of nivolumab group. Not only occurrence, but the severity (frequency of ≥G3 irAEs) was also worse in the combo therapy. Similarly, immunotherapy plus chemotherapy showed better efficacy in IMpower133 and CASPIAN study, but at the price of more irAE events. Furthermore, adding ipilimumab to this combination brought no additional benefit, but significantly higher toxicities.

The exact pathophysiology of irAEs is unclear, but the toxicity between CTLA-4 and PD-L1/PD-1 inhibitors is quite different. Pituitary cells translocate to express CTLA-4. The CTLA-4 antibody binds to the pituitary and induces lymphocyte infiltration, and tissue destruction is triggered (38, 39). PD-L1 was highly expressed on the surface of myocardial cells in two patients with immune myocarditis, leading to the recognition of myocardial and tumor surface antigens by the same T cell clone, which ultimately cause destruction of organ (16). In Keynote001 trial, 10 patients were newly diagnosed with hypothyroidism after receiving pembrolizumab, and eight of them were diagnosed with anti–thyroid antibody (40). It was suggested that irAE may be associated with autoantibodies. CTLA-4 Inhibitors reduce the number and activity of Treg cells, resulting in increased activity of TH17 cells and increased IL-17 release, contributing to the onset of immune-related colitis (41–43).

**Figure 3:**
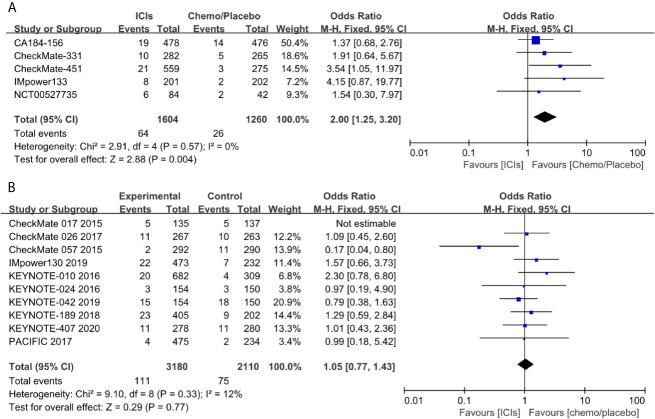
Pooled analysis of neuromuscular toxicity in SCLC **(A)** and NSCLC **(B)**.

Because SCLC is a kind of neuroendocrine tumor, also autoimmune encephalitis was frequently reported for this disease, we proposed there might be an increased occurrence of neuromuscular toxicity during the immunotherapy. To test this hypothesis, we performed a pooled analysis of the reported neuromuscular toxicity from the above trials. We found less occurrence in the control group, compared with that in immunotherapy group (**Figure 3A**). To confirm this observation, we performed a similar analysis in NSCLC trials. Conversely, immunotherapy and control groups had comparable toxicity (**Figure 3B**). This further supported the notion the neuromuscular toxicity of immunotherapy was specifically restricted in SCLC.

**Figure 4:**
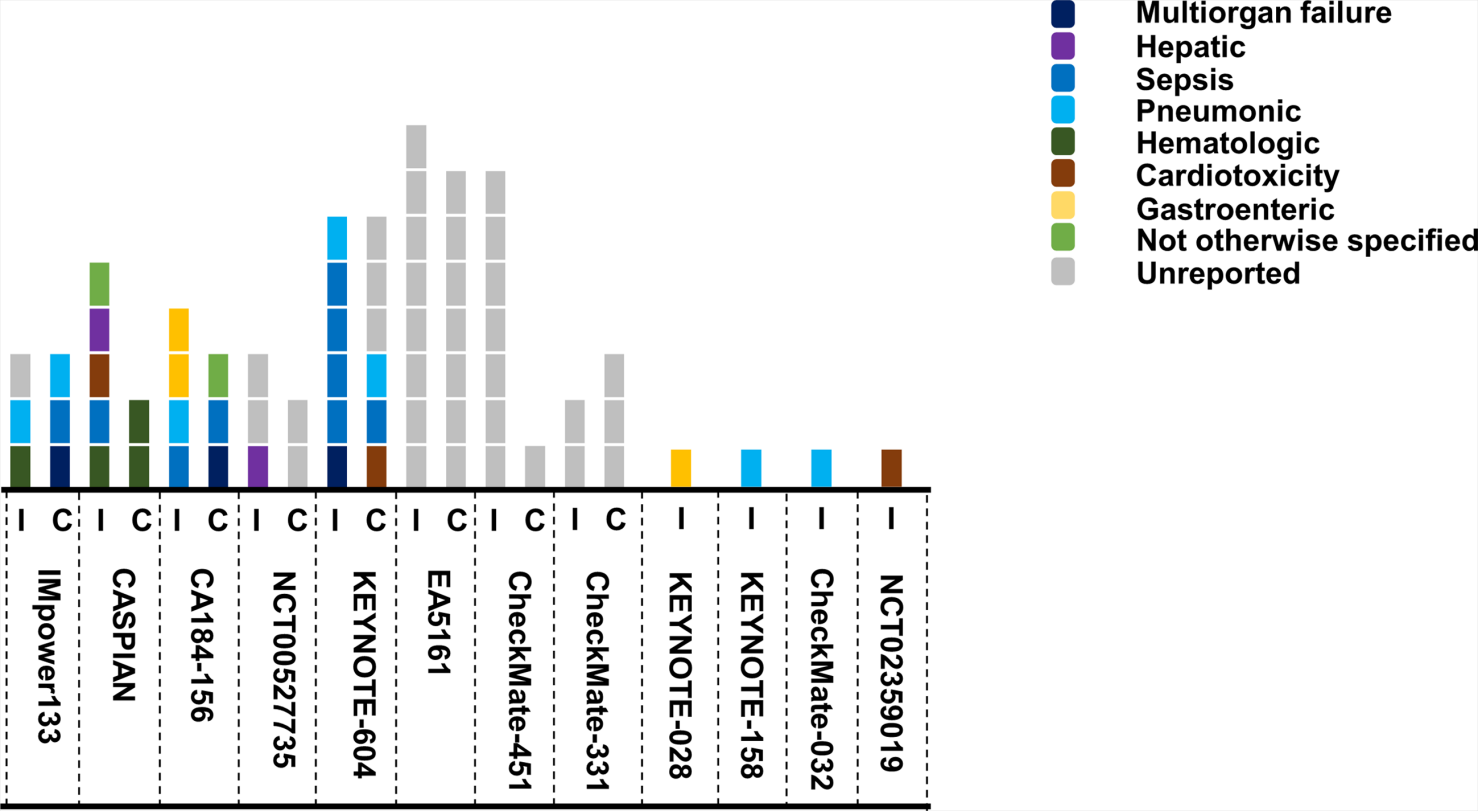
Summary of death events in SCLC trials. Each square represented one event, and different color stood for the causes of death. I, ICIs group; C, chemo/placebo group.

We paid special attention to the fatal toxicities. Immunotherapy and chemotherapy had a similar incidence of treatment-related death for SCLC patients. Totally 36 and 27 death events occurred from seven head-to-head trials respectively (**Figure 4**). From all the trials, the most common reason of reported death were sepsis (n = 7) and pneumonitis (n = 7), followed by multiorgan failure (n = 3), hematologic disease (n = 2), cardiotoxicity (n = 3), hepatitis (n = 3), and other unspecified cause (n = 2).

## Conclusion

This paper reviewed the current status of immunotherapy in SCLC. Immunotherapy brings new hope to this formidable disease, and also unprecedented toxicity profile. Immunotherapy combined with either chemotherapy or other immunotherapies, led to higher occurrence of AE than immunotherapy alone. The toxicity of immunotherapy in SCLC seemed to be different with those in NSCLC, esp. for neuromuscular toxicity. This review may be helpful for the appropriate clinical use of immunotherapy for SCLC.

## Author Contributions

Z-YD and YZ contributed conception. YF drafted the manuscript, Z-YD reviewed the manuscript, and P-PW edited the manuscript. All authors contributed to the article and approved the submitted version.

## Conflict of Interest

The authors declare that the research was conducted in the absence of any commercial or financial relationships that could be construed as a potential conflict of interest.
